# Grossesse sur cicatrice de césarienne: à propos d'un cas et revue de la literature

**DOI:** 10.11604/pamj.2015.20.122.6054

**Published:** 2015-02-12

**Authors:** Boutaina Lachiri, Abdelgheni Zazi, Zineb Benkerroum, Moulay Rachid Hafidi, Jaouad Kouach, Driss Moussaoui Rahali, Mohamed Dehayni

**Affiliations:** 1 Service de Gynécologie-Obstétrique, Hôpital Militaire d'Instruction Med V, Maroc

**Keywords:** Grossesse ectopique, grossesse sur cicatrice utérine, facteurs de risques, traitement conservateur, ectopic Pregnancy, pregnancy on uterine scar, risk factors, conservative treatment

## Abstract

La grossesse sur cicatrice de césarienne est une forme rare de grossesse ectopique qui engage le pronostic vital et fonctionnel utérin par hémorragie ou par rupture utérine précoce. Nous rapportons le cas d'une grossesse ectopique sur cicatrice de césarienne diagnostiquée à 11 semaines d'aménorrhée suite à une aspiration blanche pour grossesse arrêtée chez une patiente de 43 ans porteuse d'un utérus bi-cicatriciel. A travers cette observation ainsi qu'une revue de la littérature les auteurs vont essayer de mettre le point sur les méthodes diagnostiques et thérapeutiques de cette entité rare afin d'améliorer la prise en charge.

## Introduction

La grossesse sur cicatrice de césarienne (cesarean scar pregnancy: CSP) est définie comme une grossesse extra-utérine intégrée dans le myomètre d′une cicatrice de césarienne précédente [1]. L'incidence est estimée entre 1 /1800, et 1 / 2216 grossesses. Seules des séries limitées et des rapports de cas ont été retrouvés dans la littérature [2]. La possibilité d'une grossesse sur cicatrice de césarienne est rarement suspectée, ce qui crée la nuance dans le diagnostique comme étant une grossesse cervicale ou un avortement en cours. Ainsi, le diagnostic précoce d′une grossesse sur cicatrice de césarienne peut être retardé, et des complications potentiellement catastrophiques peuvent s′ensuivre. Nous présentons ici le cas d'une patiente qui était admise pour prise en charge d'une grossesse sur cicatrice de césarienne. Nous discuterons ensuite au vu de la littérature récente les méthodes diagnostiques et thérapeutiques à disposition pour une meilleure prise en charge des grossesses sur cicatrice de césarienne.

## Patient et observation

**Figure 1:**
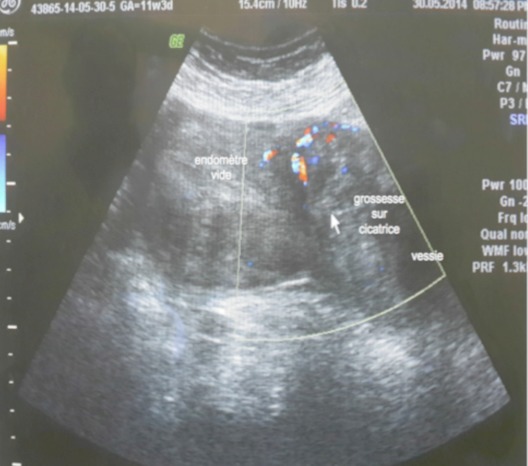
La grossesse ectopique sur la cicatrice de césarienne avec une cavité utérine vide (image échographique sur une coupe sagittale)

**Figure 2:**
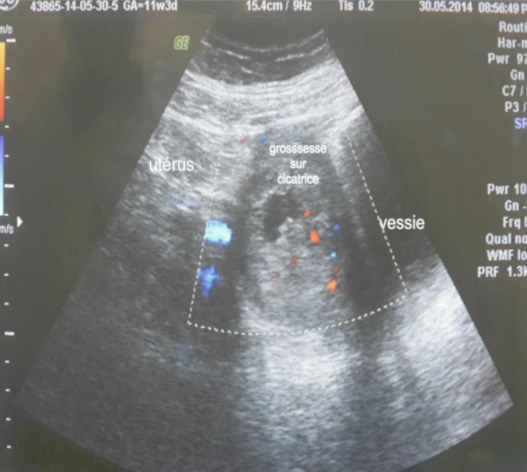
Sac gestationnel implanté sur la cicatrice de césarienne (image échographique sur une coupe transversale)

**Figure 3:**
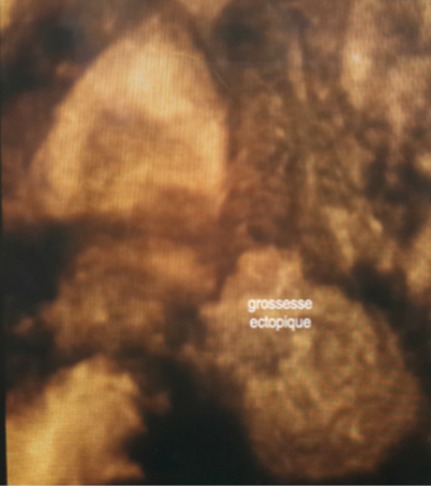
Aspect échographique en mode tridimensionnel de la grossesse sur cicatrice de césarienne

**Figure 4:**
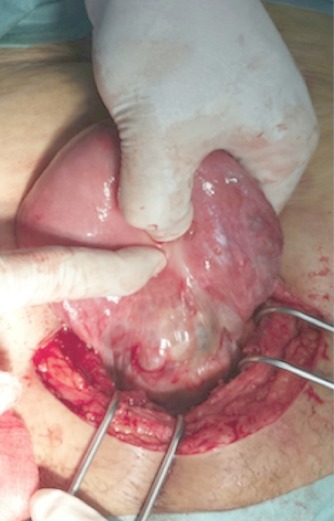
Utérus en rupture incomplète, la grossesse ectopique sur la cicatrice de césarienne est visible à travers la séreuse (image per opératoire)

**Figure 5:**
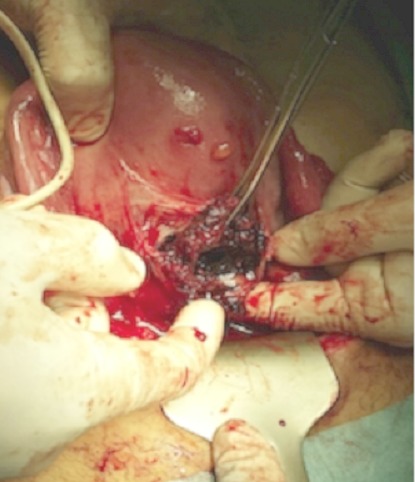
Incision utérine et résection de la grossesse ectopique (image per opératoire)

Mme M.S âgée de 43 ans, quatrième geste, deuxième pare, dans ses antécédents obstétricaux on trouve une notion d'avortement spontané à 2 mois de grossesse non cureté, deux accouchements par césarienne (la première en 2009 pour stagnation de la dilatation à 4 cm et la deuxième à 2013 pour diabète gestationnel et utérus cicatriciel) donnant naissance à deux garçons bien portants. Elle consulte aux urgences gynécologiques pour des douleurs pelviennes et métrorragies foncées de faible abondance évoluant depuis trois jours sur une aménorrhée de 11 semaines. A l'examen clinique l’état hémodynamique était stable, la palpation abdominale ne retrouvait aucun critère de gravité, l'examen au speculuma confirmé l'origine endo-utérine du saignement, et le toucher vaginal combiné au palper abdominal a objectivé un utérus augmenté de taille sans masse latéro utérine palpable ni signe d'irritation péritonéale. Une première échographie faite aux urgences avait conclu à un sac gestationnel intra-utérin festonné «bas implanté» sans écho embryon. Le taux de βHCG plasmatique est revenu à 20550 UI/ML. On a conclut à une grossesse arrêtée et la patiente a bénéficié d'une aspiration qui était blanche. Une deuxième échographie endo-vaginale réalisée a permis le diagnostic d'une grossesse isthmique avec un sac gestationnel intra-myometrial implanté sur la cicatrice de césarienne sans écho-embryon visible (Figure 1, Figure 2, Figure 3). La possibilité d'un traitement médical a été discutée mais, la patiente a présenté une aggravation de la symptomatologie avec augmentation de l'intensité des douleurs pelviennes associée à des métrorragies de moyenne abondance ayant nécessité la réalisation d'une laparotomie en urgence. A l'exploration on trouvait un utérus en rupture incomplète avec la présence d'une grossesse isthmique sur la cicatrice utérine visible sous la séreuse (Figure 4). Les opérateurs ont procédé au début à un décollement de la vessie puis ils ont réalisé une incision en regard de la grossesse ectopique qui a permis la résection du produit de conception (Figure 5) et la réfection par la suite de la cicatrice utérine. Les suites postopératoires étaient simples. L'histologie a confirmé le diagnostique d'une grossesse arrêtée. Le taux de βHCG est revenu négatif au bout d'une semaine.

## Discussion

La littérature récente suggère que la grossesse sur cicatrice de césarienne est plus fréquente qu′on ne le pensait précédemment. Son incidence est estimée entre 1/1800 et 1/2216 grossesse, et elle constitue 6.1% de toutes les grossesses extra-utérines avec une histoire d′au moins un accouchement par césarienne [3]. les facteurs de risques incriminés sont similaires à ceux du placenta accreté: d'une part, le nombre de césariennes antérieures et de gestes endo-utérins (curetages, révision utérine manuelle), d'autre part, les techniques de fécondation in vitro (FIV) avec transfert d'embryon sont également discutées dans le mécanisme [2, 4]. D'un point de vue physiopathologique, un micro-défect de la cicatrice d'hystérotomie permettrait l'invasion du muscle utérin par le blastocyste: cette hypothèse est validée par la relation qui existe entre l'indication de césarienne antérieure pour siège et le risque de CSP; ces césariennes étant souvent programmées, le segment inférieur moins sollicité et moins mature ne permettrait pas une qualité optimale de cicatrisation et favoriserait l'implantation ectopique de l’œuf [5]. Notre patiente avait un utérus bi-cicatriciel. Elle aurait une mauvaise cicatrisation. La fibrose et la faible vascularisation de la région seraient responsables d'une cicatrisation insuffisante et plus large sur un utérus multi cicatriciel [5]. Le risque d'insertion cicatricielle de l’œuf serait augmenté chez cette patiente. La clinique peut être asymptomatique. Une étude de série avait retrouvé jusqu’à 40% de patientes ne manifestant ni douleur ni saignement vaginal [2]. La rupture utérine peut être la conséquence d'un retard de diagnostic d'une grossesse sur cicatrice de césarienne, un diagnostic rapide et précis est essentiel [3]. Le diagnostic doit se baser sur les antécédents de la patiente et les manifestations cliniques, incluant les douleurs abdominales et les saignements, qui peuvent aller de simples spottings à une hémorragie mortelle. Ces hémorragies peuvent être spontanées ou iatrogènes à la suite d'un curetage. Une erreur diagnostique et une prise en charge comme fausse couche par curetage d'emblée pourraient entrainer une hémorragie massive [2]. L’échographie bidimensionnelle par voie endo-cavitaire est l'examen radiologique de première intention permettant de porter le diagnostique. Ce dernier, repose sur les critères établis par Vial en 2000 [6] associant d'abord: un utérus vide; un canal cervical vide; l'existence, sur une coupe sagittale de l'utérus, d'une disruption du sac gestationnel sur le mur utérin antérieur.

La diminution de l’épaisseur du myomètre entre le sac gestationnel et la vessie qui reflète la profondeur de l'implantation et une hyper vascularisation péri-trophoblastique objectivée par le doppler couleur ou énergie, constituent les signes échographiques indirects de CSP. En cas de doute diagnostique persistant après l’échographie, d'autres examens d'imagerie peuvent être réalisés: le mode tridimensionnel échographique ou l'IRM permettent d'appréhender les rapports anatomiques en précisant la profondeur de l'invasion trophoblastique dans le myomètre, et l'atteinte potentielle de la séreuse ou de la vessie. Si le diagnostic est évident à l’échographie bidimensionnelle, ces examens poussés ne sont pas recommandés [4]. Bien que la plupart des cas rapportés sont diagnostiqués à tort au début comme fausse couche ou grossesse cervicale, un haut niveau de suspicion clinique et à l′aide de l’échographie ces situations peuvent être facilement différenciées [7]. Dans cette observation nous rapportons un cas de CSP qui était diagnostiqué à tort comme grossesse arrêtée. Cette confusion est courante d'ou la nécessité dans la plupart des cas d'un balayage échographique répété avant de porter le diagnostic. Vu la rareté de cette situation il n'existe pas à l'heure actuelle des recommandations formelles concernant les modalités thérapeutiques. Le traitement considère l’âge gestationnel, les moyens thérapeutiques disponibles, le désir de fertilité ultérieure de la patiente, l'expérience de l’équipe thérapeutique, et les complications d'une thérapeutique de première ligne. Actuellement, le traitement, qu'il soit médical ou chirurgical, reste conservateur, sauf en cas d’échappement thérapeutique. Le traitement médical est basé sur l'administration du méthotrexate par voie locale ou systémique. Certains auteurs utilisent le chlorure de potassium injecté dans le thorax fœtal combiné au méthotrexate en injection intra-ovulaire [7]. Ce traitement nécessite une surveillance rapprochée et prolongée jusqu’à la résolution complète de la grossesse ectopique. L'aspiration-curetage est à risque hémorragique et de rupture utérine: contre-indiquée à l'aveugle, elle reste acceptable sous contrôle échographique en cas de sac gestationnel développé vers la cavité [4]. La laparoscopie et la laparotomie peuvent permettre une résection complète de la cicatrice et du tissu trophoblastique [2]. L'abord cœlioscopique tend à supplanter la laparotomie mais une grande expertise chirurgicale est nécessaire, garante d'une suture myometriale de qualité (donc solide en vue d'une grossesse ultérieure). La cœlioscopie permet enfin pour certains, de contrôler l’évacuation utérine par hystéroscopie ou simple curetage et d'associer une ligature artérielle préventive aux gestes utérins [8]. Concernant le pronostic obstétrical, des grossesses ont été décrites après une CSP. Vu le risque augmenté de rupture utérine, la césarienne programmée dès que la maturation pulmonaire est acceptable, constitue la voie d'accouchement privilégiée. Les recommandations sont de respecter un délai avant de commencer une nouvelle grossesse qui varie selon les équipes de deux mois à deux ans. Le risque de récidive de CSP existe, il est de l'ordre de 5% [9].

## Conclusion

La grossesse sur cicatrice est l'une des complications tardives de la césarienne. Son diagnostic permet de choisir une thérapeutique adaptée en fonction du plateau technique et du désir de la patiente. Le traitement de ces grossesses ectopiques doit être précoce et actif du fait du risque majeur d'hémorragie ou de rupture utérine mettant en jeu le pronostic vital et fonctionnel utérin.

## References

[1] Lai YM, Lee JD, Lee CJ, Chen TC, Soong YK (1995). An ectopoic pregnancy embedded in themyometrium of a previous cesarean section. Acta Obstet Gynecol Scand..

[2] Belinga E, Mbo AJ, Hanen C, Ali EH, Voulgaroupoulos M, Dauptain G, Cordesse A, Nko'o AS (2014). Grossesse sur cicatrice de césarienne: apport de l’échographie dans le diagnostic et la prise en charge. Health Sci Dis..

[3] Rotas MA, Haberman S, Levgur M (2006). Cesarean Scar Ectopic Pregnancies: Etiology, Diagnosis, and Management. Obstetrics & Gynecology..

[4] Maymon R, Halperin R, Mendlovic S, Schneider D, Vaknin Z, Herman A (2004). Ectopic pregnancies in cesarean section scars: the 8-years experience of one medical center. Hum Reprod..

[5] Maymon R, Halperin R, Mendlovic S, Schneider D, Herman A (2004). Ectopic pregnancies in a caesarean scar: review of the medical approach to an iatrogenic complication. Hum Reprod Update..

[6] Vial Y, Petignat P, Hohlfeld P (2000). Pregnancy in a cesarean scar. Ultrasound Obstet Gynecol..

[7] Al-NAzer Al, Omar L, Wahba M, Abbas T, Abdulkarim M (2009). Ectopic intramural developing at the site of a cesarean section scar: a case report. Cases J..

[8] Kung FT, Huang TL, Chen CW, Cheng YF (2006). Image in reproductive medicine Cesarean scar ectopic pregnancy. Fertil Steril..

[9] Ben Nagi J, Helmy S, Ofili-Yebovi D, Yazbek J, Sawyer E, Jurkovic D (2007). Reproductive outcomes of women with a previous history of caesarean scar ectopic pregnancies. Hum Reprod..

